# Personalized regenerative strategies and molecular diagnosis for *in vitro* fertilization success: a case report

**DOI:** 10.1093/omcr/omad037

**Published:** 2023-04-20

**Authors:** Dinorah Hernández-Melchor, Iván Madrazo, América Padilla-Viveros, Felipe Camargo, Esther López-Bayghen

**Affiliations:** Science, Technology and Society Program, Centro de Investigación y de Estudios Avanzados del Instituto Politécnico Nacional (CINVESTAV-IPN), México City 07360, México; Instituto Regenera SC, México City 05320, México; Investigación Clínica, Instituto de Infertilidad y Genética México SC, INGENES, México City 05320, México; Science, Technology and Society Program, Centro de Investigación y de Estudios Avanzados del Instituto Politécnico Nacional (CINVESTAV-IPN), México City 07360, México; Investigación Clínica, Instituto de Infertilidad y Genética México SC, INGENES, México City 05320, México; Departamento de Toxicología, Centro de Investigación y de Estudios Avanzados del Instituto Politécnico Nacional (CINVESTAV-IPN), Av. IPN 2508 San Pedro Zac, CDMX 07360, México

## Abstract

Limited options are available for infertility associated with damaged or suboptimal tissues, typically the endometrium or ovaries. The goal of regenerative medicine is to restore function to specific tissues. Here, a 35-year-old female patient underwent two interventions of regenerative medicine: (i) autologous mesenchymal stem cells (MSCs) were applied in the myometrium, and (ii) intraovarian infusion of platelet-rich plasma (PRP). After two failed *in vitro* fertilization cycles (IVF), in which the endometrium was <5 mm, MSCs were applied, achieving a 7 mm trilaminar lining; however, the embryo quality remained poor. Therefore, intraovarian PRP was utilized for the next IVF cycle; the patient’s response improved, and a euploid embryo developed. After the embryo transfer and a normal 38 weeks of pregnancy, a baby girl was born. Here, we demonstrate two forms of regenerative medicine that can be utilized to improve IVF.

## INTRODUCTION

Assisted Reproductive Technology (ART) success in achieving a viable pregnancy depends on competent gametes to generate healthy embryos and an adequate uterus. When either of these conditions is insufficient due to suboptimal tissue function or damage, the chances of achieving pregnancy diminish. Regenerative medicine aims to restore lost function in damaged tissues. Regenerative medicine, stem cell treatments, cartilage regeneration, platelet-rich plasma (PRP) and prolotherapy has shown promise in hematopoietic development and lung [1]; therefore, it has been posited that regenerative medicine could aid in (ART) [2, 3]. Here, a patient that underwent a combination of regenerative interventions using autologous cellular products to improve endometrial quality and ovarian function and, therefore, achieve a healthy pregnancy is presented.

## CASE REPORT

A 35-year-old female patient with a 39-year-old healthy male partner was diagnosed with 10-year secondary infertility with one previous abortion and three failed artificial inseminations. Around 2 years before trying to conceive (2018), the patient suffered from uterine myomatosis and was treated by myomectomy with laparoscopy, hysteroscopy and open surgery procedures. The patient was counseled for IVF, and before the first cycle, a hysterosonography was performed to rule out synechiae.

The patient underwent four courses of controlled ovarian stimulation with a gonadotrophin-releasing hormone antagonist for 9 days (Table 1). After each oocyte retrieval, oocytes were inseminated by intracytoplasmic injection. Preimplantation Genetic Test for Aneuploidies (PGT-A) was performed following the standardized protocol with trophectoderm biopsies from good-quality embryos before vitrification; pregnancy was determined by serum beta-human chorionic gonadotropin (β-hCG) >10 mIU/mL on Day 14.

**Table 1:** Results from IVF cycles.

Cycle	1	2	3	4
Patient’s age	35	36	37	38
Surgical/regenerative intervention before ovarian stimulation	None	Hysteroscopy (normal cavity)	Autologous MSC trans-myometrial implantation (47.3 × 10^7^ live cells)	Bilateral intraovarian PRP infusion
E_2_ (mg/dl), Day 10	2200	1885	1167	1333
Retrieved oocytes	12	10	6	8
Fertilized embryos/Quality (inner cells mass and trophectoderm)	Three; Day 5/BC	One; Day 6/CB	One; Day 5/BC	One; Day 5/BC One; Day 6/BC
PGT-A results	(i) Euploid XY (ii) Aneuploid, T19, XY (iii) Aneuploid, T19 & T16, XX	(i) Euploid, XY	(i) Aneuploid M22, XY	(i) Aneuploid, T19, XY (ii) Euploid, XX
Frozen–thawed embryo transferred	One; Day 5	One; Day 6	None	One; Day 6
Endometrial thickness	<5 mm	<5 mm	7 mm	8 mm
β-hCG (mU/dL)	0.01	0	0.1	82.5
				Normal pregnancy, healthy baby girl, 38 weeks, Apgar 8

The initial two IVF cycles were ideal concerning the number of oocytes retrieved, estradiol (E2) levels on Day 2 and fertilization rate; endometrial preparation for embryo transfer was performed during an estrogen-primed cycle; however, the transfer of one euploid embryo failed (Table 1). During the second IVF cycle, the uterus was determined to be normal by hysteroscopy, but the embryo failed to implant.

**Figure 1:**
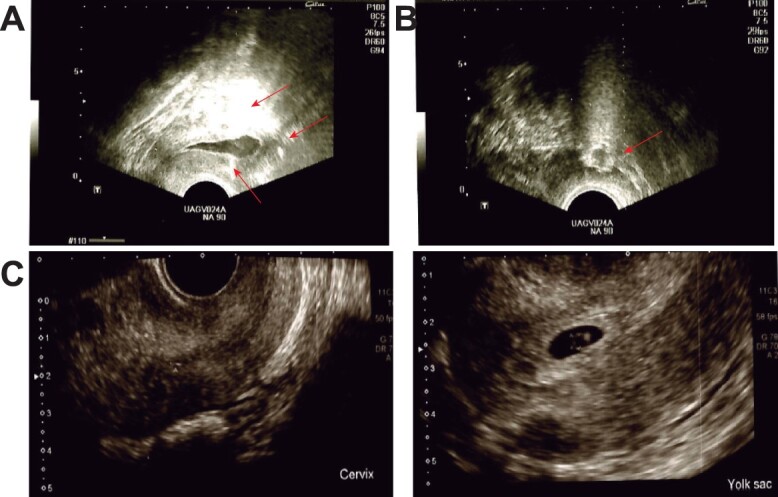
Personalized regenerative interventions for ART success. (**A**) Trans-myometrial injection of MSC. The procedure was performed under intravenous anesthesia and antibiotic cover, with the patient laid in a lithotomy position. Under transvaginal ultrasound guidance, the sub-endometrial zone was located and a 35 cm 17 G single-lumen needle was inserted vaginally, and 3 mL of the autologous cell suspension at the fundus, anterior and posterior part of the uterine cavity at the myometrium layer; (**B**) Intraovarian infusion of PRP. PRP bilateral injection was performed during the late follicular phase of the menstrual cycle under transvaginal ultrasound guidance and sedation anesthesia. The ovaries were aligned with the 35 cm 17 G single-lumen needle to avoid intervening vascular or other structures; the needle was quickly advanced without rotation deep into the central ovary. Once tip placement was confirmed, the PRP was slowly injected (2 ml) in each ovary within the ovarian cortex; (**C**) Ultrasound to ensure uterine implantation of single gestational sac after the proposed surgical interventions; the sonograms are at 8 weeks after embryo transfer.

For the third IVF cycle, the first regenerative medicine intervention was suggested to the patient to address endometrial thickness; adipose tissue obtained by micro liposuction was washed, mechanically disaggregated and treated with collagenase type I to isolate autologous mesenchymal stem cells (MSC) in the stromal vascular fraction (SVF). Again, isolated cells were counted, and trans-myometrialy injected into the patient’s uterus to improve endometrial quality (Fig. 1). Even when endometrial thickness improved, no euploid embryos were available.

For the fourth cycle, to enhance ovarian function, autologous PRP was isolated from venous blood by centrifugation and bilaterally injected within the ovarian cortex under sedation anesthesia (Fig. 1). One euploid embryo was transferred, and implantation was confirmed by β-hCG (Table 1). Afterward, the embryo sac was located via ultrasound on week 7. After a normal pregnancy, at 38 weeks, a healthy baby girl was born (Apgar 8).

## DISCUSSION

Regenerative medicine aims to restore the structure and function of damaged tissues. Therefore, this strategy may help rehabilitate ovarian and endometrial tissues in infertile patients. Indeed, combining surgical interventions using autologous cellular products to improve endometrial quality and ovarian function, coupled with IVF and molecular diagnosis of aneuploidy, was a powerful combination to achieve a healthy pregnancy in the infertile female patient.

One limitation for successful IVF results is the need for good ova to produce healthy embryos. To overcome this, some patients accept ova from donors; however, options remain limited for patients unwilling to accept donations. PRP is a concentration of human platelets obtained from the patient’s blood that is 5- to 10-fold higher than the physiologic concentration of thrombocytes in the whole blood and contains a variety of growth factors and bioactive molecules involved in clotting, inflammation, cell growth, cell adhesion and host defense [4]. Here, intraovarian infusion of PRP improved the number of antral follicles, estradiol production and collected oocytes. Consistent with the case reports reviewed by Atkinson *et al.* [4], where using PRP on ovaries demonstrated improvements in ova quantity and quality as well as embryo availability, clinical pregnancy and live birth rates, in which the proposed mechanism involves cytokines, such as BMPs, EGF, PDGF, TGF- βI and SIP [4]. However, improving the oocyte and embryo quality may not be sufficient to overcome a poor endometrium; this means targeting multiple tissues to achieve pregnancy may be necessary.

A key characteristic for optimal implantation is the presence of a trilaminar endometrium with a minimum thickness of 7 mm [5]. In patients with recurrent implantation failure or thin endometrium, the intrauterine infusion of PRP improved endometrial thickness [6]. This effect seems to involve growth factors, such as the Epidermal Growth Factor (EGF), Transformation Growth Factor Beta (TGF-β), and Vascular Endothelial Growth Factor (VEGF), and specific cytokines, such as Leukemia Inhibitory Factor (LIF) and Interleukins-1, −6, −11, and − 15, known to regulate inflammation [7]. While the optimal stem cell type for a specific application may vary, MSCs, such as hematopoietic, non-hematopoietic and bone marrow-derived stem cells, can increase the presence of growth factors and cytokines, resulting in clinical benefits to reconstitute the endometrial tissue architecture and allow to increase the endometrial thickness as reviewed by Zhao *et al.* [8]. Here, as the source for MSC, we used SVF, the minimum manipulated heterogeneous cell population isolated from the adipose tissue with comparable regenerative potential as cultured MSCs. SVF contains MSCs, endothelial precursors, T-regulatory and smooth muscle cells, macrophages, pericytes and preadipocytes [9]. The benefits of SVF are accessibility and minimal manipulation with a higher yield of live cells. Inducing conditions for marrow-derived versus fat-derived MSCs may result in different microenvironments [10]. MSC from the SVF can be considered for certain patients with multiple causes of infertility, known or unknown; as shown here, two consecutive forms of regenerative medicine could improve IVF outcomes.

Personalized regenerative strategies coupled with the systematic molecular diagnosis of aneuploidy are a powerful combination to improve endometrial quality, ovarian function and embryo selection to achieve pregnancy in infertile patients with their ova. In addition, the variety of personalized regenerative strategies added to ART offers alternatives to manage dysfunctions in female reproductive tissues and achieve pregnancy.
